# Behavior policy learning: Learning multi-stage tasks *via* solution sketches and model-based controllers

**DOI:** 10.3389/frobt.2022.974537

**Published:** 2022-10-12

**Authors:** Konstantinos Tsinganos, Konstantinos Chatzilygeroudis, Denis Hadjivelichkov, Theodoros Komninos, Evangelos Dermatas, Dimitrios Kanoulas

**Affiliations:** ^1^ Department of Computer Engineering and Informatics (CEID), University of Patras, Patras, Greece; ^2^ Computer Technology Institute and Press “Diophantus” (CTI), Patras, Greece; ^3^ Centre for Artificial Intelligence, University College London (UCL), London, United Kingdom; ^4^ Department of Computer Science, University College London (UCL), London, United Kingdom

**Keywords:** imitation learning, multi-stage tasks, evolutionary strategies, reinforcement learning, sim2real

## Abstract

Multi-stage tasks are a challenge for reinforcement learning methods, and require either specific task knowledge (e.g., task segmentation) or big amount of interaction times to be learned. In this paper, we propose Behavior Policy Learning (BPL) that effectively combines 1) only few solution sketches, that is demonstrations without the actions, but only the states, 2) model-based controllers, and 3) simulations to effectively solve multi-stage tasks without strong knowledge about the underlying task. Our main intuition is that solution sketches alone can provide strong data for learning a high-level trajectory by imitation, and model-based controllers can be used to follow this trajectory (we call it behavior) effectively. Finally, we utilize robotic simulations to further improve the policy and make it robust in a Sim2Real style. We evaluate our method in simulation with a robotic manipulator that has to perform two tasks with variations: 1) grasp a box and place it in a basket, and 2) re-place a book on a different level within a bookcase. We also validate the Sim2Real capabilities of our method by performing real-world experiments and realistic simulated experiments where the objects are tracked through an RGB-D camera for the first task.

## 1 Introduction and related work

Reinforcement learning (RL) (Sutton and Barto, 1998) provides a solid theoretical framework that can give the ability to robotic systems to learn by trial-and-error. Recently, there exists a renewed interest in RL in the robotics literature mainly driven by the recent successes of deep learning (LeCun et al., 2015). A few success highlights outside of robotics include RL-based agents that play many of the Atari 2,600 games better than humans, or that can beat the world’s best human players at Go and chess with minimal human hard-coded knowledge (Silver et al., 2017). These impressive results, however, are difficult to achieve in robotics applications mainly due to the data-hungry nature of the RL algorithms (Chatzilygeroudis et al., 2019): for example, 4.8 million games were required to learn to play Go from scratch (Silver et al., 2017), 38 days of play (real time) for Atari 2,600 games (Mnih et al., 2015), and, for example, about 100 h of simulation time (much more for real time) for a 9-DOF mannequin that learns to walk (Heess et al., 2017).

By contrast, robotic applications are on the other end of the spectrum and have to face the micro-data challenge, that is to learn by trial and error in a handful of trials (Chatzilygeroudis et al., 2019). When learning with a physical robotic mechanism, minimizing the interaction time is crucial. This is mainly because: 1) the real world cannot be accelerated or parallelized, 2) the physical robots can be damaged (RL algorithms often try “unsafe” behaviors especially in the beginning of the training), 3) the engineering work required to keep a robot running for long periods of time is expensive, and 4) adaptation to novel situations is only useful if it can be done in reasonable time.

Learning from demonstrations (LfD) (Billard et al., 2008; Stulp and Sigaud, 2013; Vecerik et al., 2017) is a powerful technique to accelerate learning on robotics systems. The main idea behind it is to utilize expert (possibly human) demonstrations about the task in order to bootstrap the learning. Using this type of approaches, robotic manipulators can learn to draw digits or perform complex trajectories using only a few demonstrations (Khansari-Zadeh and Billard, 2011), or even humanoids can learn how to navigate and co-manipulate objects (Figueroa et al., 2020). LfD methods usually rely on structured policies and model-based low-level controllers (Chatzilygeroudis et al., 2019; Liang and Boularias, 2021). Another promising direction is using robotic simulators to learn robust policies that can generalize to many different variations of physical/task properties (Tobin et al., 2017) or to bootstrap learning with good initialization (Cutler and How, 2015). Sim2Real methods, as they are usually referred to, have provided a wide range of successful applications in robotic systems, ranging from manipulation tasks (James et al., 2017; Chebotar et al., 2018; Peng et al., 2018) to multi-leg locomotion problems (Chatzilygeroudis and Mouret, 2018; Tan et al., 2018; Hwangbo et al., 2019; Lee et al., 2020).

Despite the successes of the above techniques, there are a few limitations that prohibit their wide adoption in practical real applications. Most LfD approaches require the knowledge of the “optimal” actions/commands (Vecerik et al., 2017; Rajeswaran et al., 2018) (or even policies (Ross et al., 2011)), which makes it necessary to actually control the robot while performing the demonstrations. This can be difficult to be done in safe-critical tasks and collecting this type of demonstrations requires more effort than collecting solution sketches either *via* kinesthetic guidance or a GUI. Moreover, LfD approaches that operate in the task-space usually require strong knowledge of the underlying task and work best for point-to-point motions (Billard et al., 2008; Khansari-Zadeh and Billard, 2011; Bahl et al., 2020; Pirk et al., 2020). This practically means that we need to split the task in subtasks by hand and provide the algorithm with segmented data for it to work reliably. Moreover, most successful approaches learn “reaction” policies, that is policies that do not perform long-term planning, but react quickly to what they see (Lee et al., 2020). Lastly, while there exist a few approaches that attempt to learn with visual observations (i.e., images) (Levine et al., 2016; James et al., 2017; Mandlekar et al., 2021), they usually require a large amount of examples that make it difficult to use them in practical applications.

At the moment and to the best of our knowledge, no practical imitation learning can learn how to solve multi-stage tasks by utilizing only solution sketches and little to no interaction with the physical robot. There are even fewer successful methods that rely solely on vision sensors. In essence, the most successful algorithms utilize external camera systems (e.g., motion capture systems) to infer the state of the environment or require big amount of interaction time, otherwise.

**FIGURE 1:**
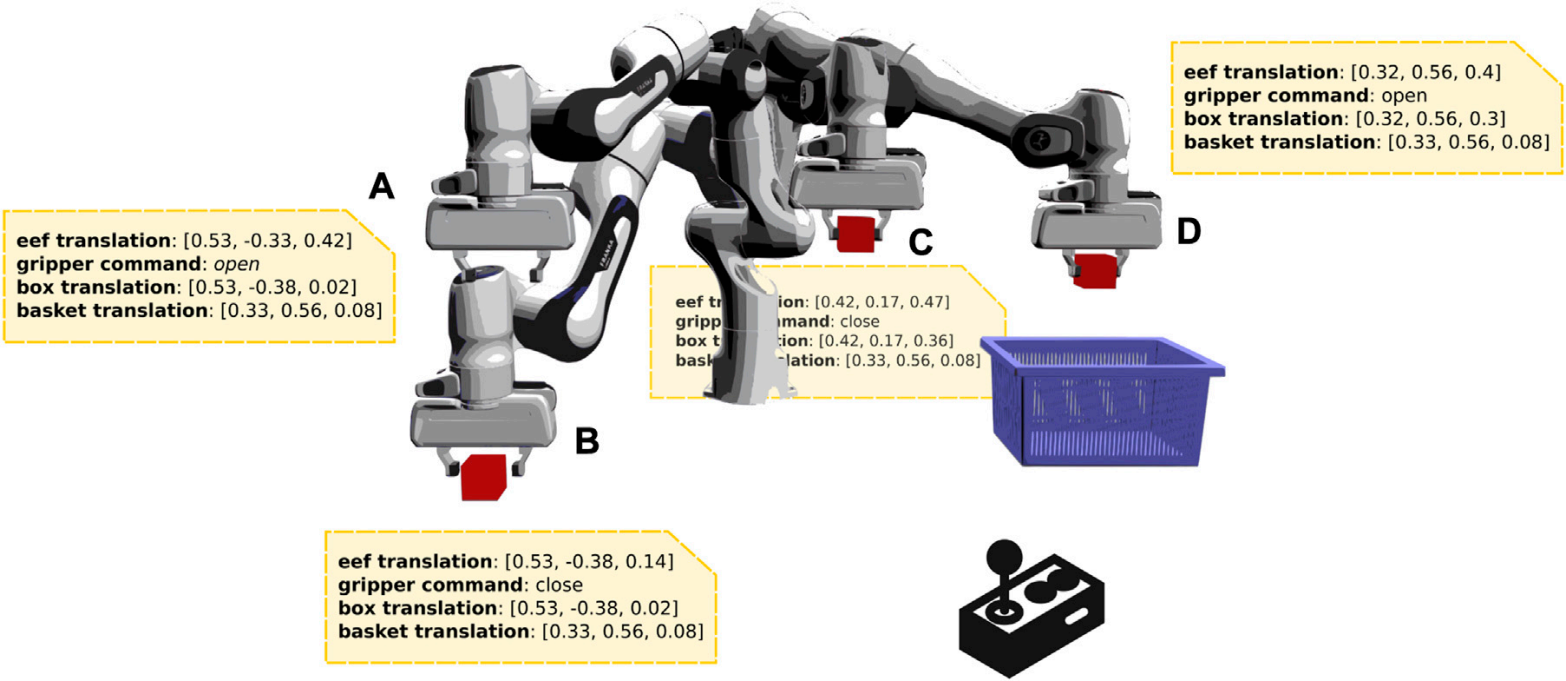
An example of a solution sketch. The end-effector of a manipulator is controlled via a joystick to solve a pick and place task. High-level information of the trajectory followed (e.g., robot and object states), consisting only of state variables, constitutes a solution sketch. As an example, the states marked **(A–D)** in the figure showcase potential points on the trajectory with corresponding state information.

In this paper, we refer to solution sketches as demonstrations consisting only of robot and environment state variables, but no control commands. They are practically easy to be acquired through kinesthetic guidance or a joystick (Figure 1), as there is no need to program a controller for solving the task. These sketches provide high-level information of the trajectory followed while performing the task and of the state of the environmental task-related objects as well. Thus, solution sketches provide environment-specific information, as we know the objects to be handled, but they do not require specific information of the stages of the underlying task or the controller to solve it. In this work, we propose a novel pipeline that attempts to provide a practical approach that can tackle multi-stage tasks effectively while having access only to visual information about the environment, a simulator, and few solution sketches.

**FIGURE 2:**
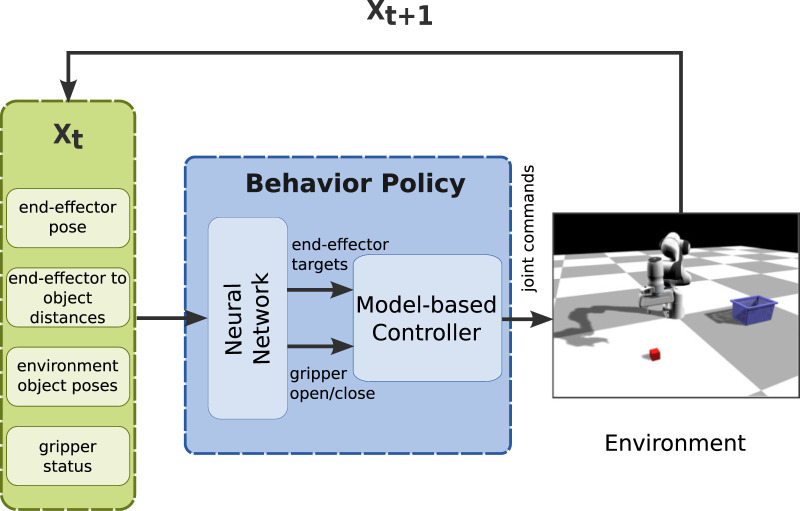
Overview of the proposed policy structure, where input includes environment-specific information (end-effector pose, end-effector to object distance, environment object poses, gripper status) and outputs the desired trajectory (joint commands), using a neural network and a model-based controller as the Behavior Policy Learning (BPL).

We take inspiration from the LfD literature (Billard et al., 2008; Khansari-Zadeh and Billard, 2011) and define a structured policy that encodes the desired trajectory to solve the task, but also takes as input environment-specific information (Figure 2). Instead of modeling the trajectory *via* dynamical systems or waypoints, we use neural networks in order to provide the learning pipeline with more flexibility. The goal of our work is to provide a practical pipeline for tackling the challenge of learning multi-stage tasks from a few solution sketches with realistic assumptions and observation spaces.

Overall, the main contributions of this work are:

• Novel policy structure: we define a policy that takes as input environment-specific data (e.g., distances to objects), but not task-specific (i.e., we do not need to have a controller that solves the task), and encodes the desired trajectory;
• Further validation of the need for structured policies and that model-based low-level controllers are essential for fast and stable learning;
• Practical approach that can learn with less than 50 solution sketches and zero interaction with the physical robot.

Using our novel pipeline we were able to successfully learn two multi-stage tasks. In particular, using our approach a robotic manipulator was able to learn how to 1) grasp a box, place it inside a basket and adapt to many unseen initial positions of the box and the basket, and 2) grasp a book from a book shelf, place it at a goal position in the top shelf of the bookcase and adapt to unseen initial positions of the book. We evaluate our approach in simulation and show that we outperform classical policy structures, and achieve similar performance with approaches that require extensive task knowledge. For the first task, we evaluate our method both with ground truth knowledge about the objects but also with a perception module to emulate the reality gap. We also present preliminary results of our method in a physical robot that has to perform the first task to showcase the Sim2Real capabilities of our method.

## 2 Proposed method

### 2.1 Behavior Policy Learning (BPL)

The key aspects of our proposed method, called Behavior Policy Learning (BPL), are as follows:

• We collect a small number of solution sketches and create a supervised learning problem where the task is to reproduce the demonstrated trajectories;
• We devise a novel policy structure in order to capture the task variations and generalize effectively;
• We use the RL policy search formulation coupled with domain randomization to further optimize the policy in a realistic simulator.

### 2.2 Policy structure

We model the robot and the environment jointly as a discrete-time dynamical system that can be described by transition dynamics of the form (deterministic dynamics and Gaussian system noise):

$$x_{t+1}=f\left(x_{t},u_{t}\right)+w$$ (1)

where the robot is at state $x_{t}\in R^{E}$ at time *t*, takes control input $u_{t}\in R^{F}$ and ends up at state ***x***_*t*+1_ at time *t* + 1, ***w*** is i. i.d. Gaussian system noise, and *f* is a function that describes the unknown transition dynamics. We assume that the system is controlled through a parameterized *policy* *π*(***u*** | ***x***, ***θ***) that is followed for *T* steps (***θ*** are the parameters of the policy). In this paper, we use *deterministic policies*; a deterministic policy means that *π*(***u*** | ***x***, ***θ***) ⇒ ***u*** = *π*(***x*** | ***θ***).

We make the assumption that the state of the system can be split into two parts: 1) a part that can be directly controlled (e.g., positions and velocities of the end-effector), and 2) a part that can only be observed (e.g., obstacles/objects). In particular (we omit the time notation, *t*, for clarity):

$$x=\left[\begin{array}{c} x^{c}\\ x^{nc} \end{array}\right]\in R^{d_{c}+d_{nc}},$$ (2)

where ***x*** ^*c*^ is the part of the state that can be directly controlled and ***x*** ^*nc*^ is the part of the state that can only be observed. *d*_*c*_ and *d*_*nc*_ are the state-space dimensions for the controllable and non-controllable parts, respectively.

This formulation allows us to create an interesting set of policy structures; one of them we describe here. In the general case, we assume that ***x*** ^*c*^ is any function of the full robot state, and ***x*** ^*nc*^ can be any function that corresponds to the observed objects. The former is usually accessible directly from the sensors of the robot or its model, whereas the latter can be done through a computer vision module (e.g., end-to-end object tracking).

In the experiments of this paper, we assume that ***x*** ^*c*^ contains only the positions of the controllable system (i.e., no velocities), that is the 3D position of the end-effector of our manipulator and the gripper status (how open it is), and ***x*** ^*nc*^ is a concatenation of all the 3D positions and distances from a fixed point on the robot to the objects (of interest) in the environment. For example, in a pick and place task, ***x*** ^*nc*^ is a 8D vector containing the 3D positions of the object to pick and to the basket/drawer we need to put it in/on and their distances to the end-effector.

The goal of BPL is to encode the desired trajectory that the robot needs to follow. For this reason, we use the future (controllable) states $x_{t+k}^{c}$ as the output of the policy (*k* > 0). In essence, the policy predicts the next target for the low-level controller at each timestep, which corresponds roughly in learning the trajectory the robot has to follow to complete the task (Figure 2). The parameters of the low-level controller can be jointly learned with the rest of the policy parameters (Gupta et al., 2019) but this is outside the scope of this work. Moreover, our work provides strong evidence that using model-based low-level controllers makes learning more stable and effective.

### 2.3 Learning from solution sketches

The policy structure defined in the previous section can be used for pure RL, but also for learning from demonstrations. In this paper, we will focus on the latter. We devise the following setup:

• We collect solution sketches, that is demonstrations containing only state variables, and no control inputs;
• We create a dataset of the form $x_{t}\to x_{t+k}^{c}$ with *k* > 0;
• We use neural networks to parameterize the policy and learn a deterministic policy that takes as input all the state variables, and outputs the next target for the controllable variables: $\pi \left(x_{t}|\theta \right)\to x_{t+k}^{c}$ .

In our experiments, we set *k* = 10 and use small neural networks with 2 hidden layers for the policy. The pipeline was quite robust to the choice of *k* and values between 1 and 15 were working similarly. Unlike previous approaches, we do not need many demonstrations and we could achieve reasonable results with as few as 10 demonstrations (Section 3). The minimum number of demonstrations depends on the task specifications. For example, if we are learning how to replicate a single trajectory we can greatly decrease the number of demonstrations. On the other hand, learning the pick and place task as described in Section 3.1, where the position of the object and the basket varies a lot, we need a few more demonstrations (i.e., with 50 demonstrations we got a good balance between the number of demonstrations and quality of results).

Our approach can be used in high-level industrial settings, where the objects of interest and vague task specifications are known. For instance, we expect to know that we need to handle a box in a pick and place scenario and also the boundaries of possible configurations of the box. We foresee a use-case where a human operator moves the robot with a joystick in a kinematic simulator (with basic object interactions) and performs several solution sketches. The initial configurations for collecting the solution sketches are evenly spaced using a Centroidal Voronoi Tesselation (CVT) (Du et al., 1999) of the space. Practically, the user gives a desired number of demonstrations, and the algorithm returns the generated initial configurations that approximate a CVT (Vassiliades et al., 2017).

### 2.4 Policy improvement using a simulator

The policy learned from the solution sketches is already effective (see Section 3.2), but our formalization allows for further fine-tuning *via* RL. Our goal is to be able to use the optimized policy directly to the physical world. In this paper, we experiment with domain randomization (Tobin et al., 2017) to robustify the policy to verify the idea. We frame the policy search optimization as a black-box optimization, and seek the maximization of a reward function *J*(***θ***) only by using measurements of the function. In this paper, we assume that the reward function returns **sparse rewards**, and *the agent gets 1 if it succeeds in solving the task, and 0 otherwise*. This type of rewards is difficult for RL algorithms to optimize (Kaushik et al., 2018), but quite intuitive for humans and easy to automate. We use Covariance Matrix Adaptation Evolution Strategy (CMA-ES) (Hansen, 2006a), which is a stochastic, derivative-free method for numerical optimization of non-linear or non-convex continuous optimization problems and has been successfully used in RL settings (Chatzilygeroudis et al., 2019, 2017). In short, CMA-ES models a population of points as a multivariate normal distribution and performs the following steps at each generation *k* (we defer to Hansen (2006b) for more details):

1) Sample *λ* new offspring according to a multi-variate Gaussian distribution of mean ***m***_*k*_ and covariance $\sigma_{k}^{2}C_{k}$ , that is, $\theta_{i}∼N\left(m_{k},\sigma_{k}^{2}C_{k}\right)$ for *i* ∈ 1, … , *λ*;
2) Rank the *λ* sampled candidates based on their performance *J*(***θ***_*i*_) and select the fittest *μ* individuals with *μ* ≤ *λ*;
3) To reflect the distribution of the *μ* best candidates, compute ***m***_*k*+1_ by averaging the *μ* individuals: $m_{k+1}=\frac{1}{\mu }\sum_{i=1}^{\mu }\theta_{i}$ , and $\sigma_{k+1}^{2}C_{k+1}$ .

In the preliminary experiments, each candidate policy was evaluated multiple times to reduce the variance. In each evaluation we spawn a new initial configuration and add small uniform noise to the observations. The noise is added to emulate the noisy observations that would come from a realistic object detection pipeline: thus we perform a type of domain randomization. Each candidate policy returns the average reward of the multiple evaluations which is computing the success rate. It is important to note that one can use any other state-of-the-art policy search algorithm as PPO (Schulman et al., 2017) or TD3 (Fujimoto et al., 2018); we chose CMA-ES because our policies are relatively low-dimensional, CMA-ES is easier to tune, in low-dimensional regimes performs adequately and is effective in policy fine-tuning (Stulp and Sigaud, 2013). We perform only a few iterations of CMA-ES with a small initial sigma (e.g. 1e − 3), as we only need to fine-tune the policy and not attempt to find a novel one.

## 3 Experimental results

In this paper, we deal mainly with manipulators and thus a natural choice for a low-level controller can be: 1) joint-space PID controller or 2) task-space PID controller. In our experiments, we use a task-space PID controller and transform the commands to the joint-space using the pseudo-inverse of the jacobian $\left(J_{b}^{†}\right)$ for the end-effector control and a joint-space PI controller for the gripper. On the physical system, we use an impedance Cartesian controller for the end-effector control. If one has to deal with more complex robotic systems, they can use any low-level model-based controller that is suited. For example, if we want to learn with a humanoid robot, one can utilize a Quadratic-Programming (QP) based low-level controller (Escande et al., 2014). We perform experiments in simulation with a Franka Panda manipulator in two tasks and provide preliminary experiments with a physical setup. We aim at answering the following questions:

1) How well does the proposed BPL perform at imitation learning? How well does it generalize to variations/unseen situations?
2) How does our proposed BPL compare to task-, robot-agnostic policies (e.g., policies that aim to replicate the optimal actions)?
3) How does our proposed BPL compare to task-specific policies (i.e., policies that utilize more task knowledge)?
4) How well does the whole BPL approach work in novel and realistic scenarios (i.e., physical robot or simulation with perception module)?

To answer to the above questions, we devise two different scenarios: 1) a scenario where the manipulator has to pick up a box and put it inside a basket, and 2) a scenario where the manipulator has to grasp a book from a book shelf, and place it at a goal position in the top self of the bookcase.

We perform two sets of experiments. In the first set, we collect a few solution sketches and evaluate the imitation learning capabilities of our approach. In essence, we do not fine-tune the policy and we perform only the supervised learning part of the method. Here we compare our BPL structure with other policy structures. In the second set of experiments, we evaluate the whole pipeline and how well it can transfer to more realistic settings. First, we evaluate the improvement of the policies in a simulated environment where the robot is not given the ground truth locations of the objects, but they are inferred *via* a simulated RGB-D sensor. Lastly, we provide preliminary experiments with a physical setup where the low-level controller, and the objects are different from the ones used in simulation.

### 3.1 Experimental setup/tasks

For each of the two tasks, we created a simulated environment using the DART simulator (Lee et al., 2018). Both environments consist of a 7-DoF Franka Panda manipulator with a gripper and other environmental objects related to the task.

#### 3.1.1 Pick and place task

**FIGURE 3:**
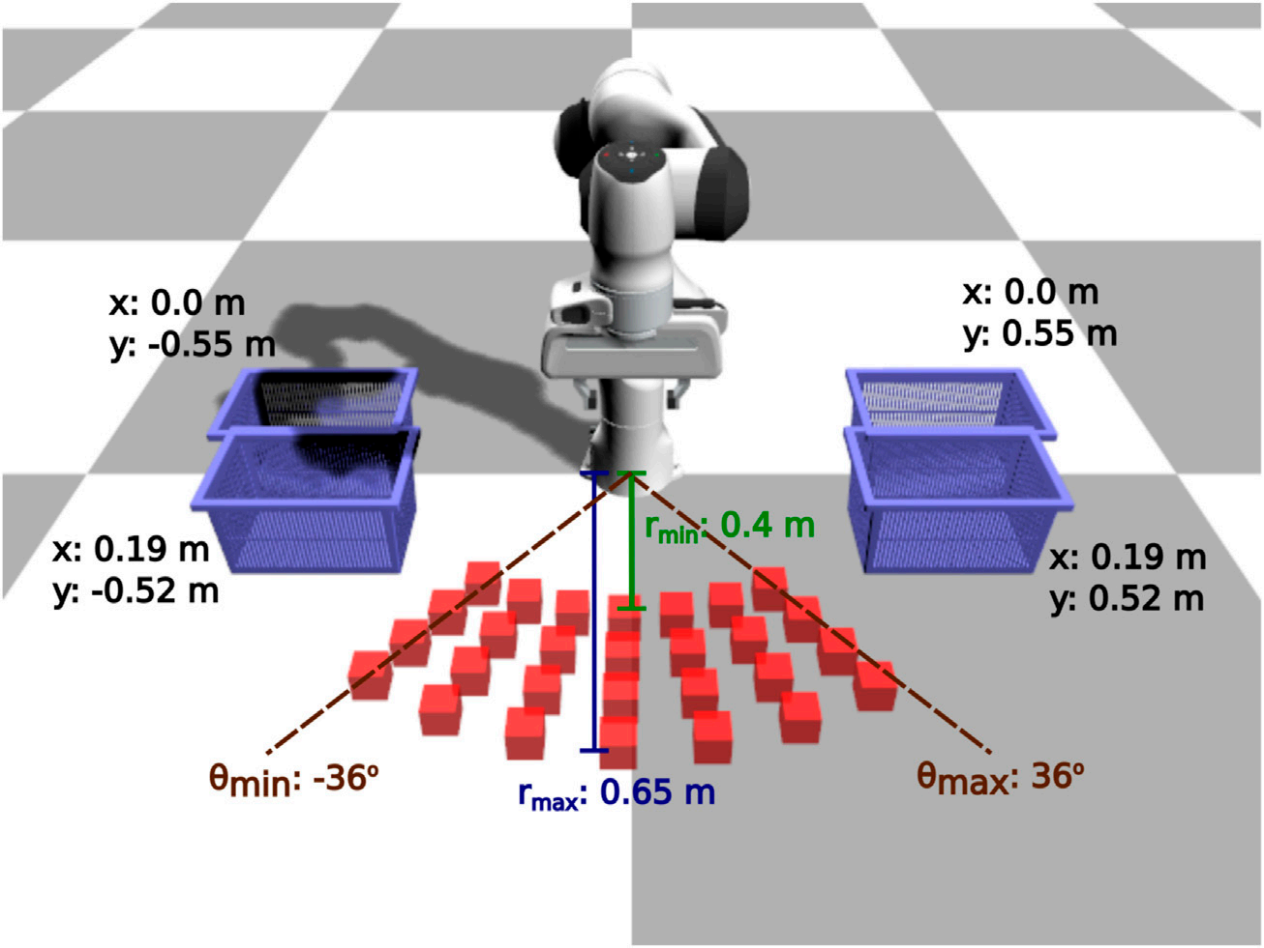
Pick and place task: evaluation configurations. We use 112 different initial configurations to evaluate the learned policies: 28 different box positions for 4 different basket positions.

In this scenario, the environment consists of the manipulator, a box and a basket, where the positions of the box and the basket vary (see Figures 2, 3). The manipulator has to perform the following sequence of sub-tasks: 1) go above the box, 2) grasp the box, 3) lift up the box, 4) go above the basket (with the gripper closed), and 5) release the box. We provide extensive results on this task.

#### 3.1.2 Bookcase task

**FIGURE 4:**
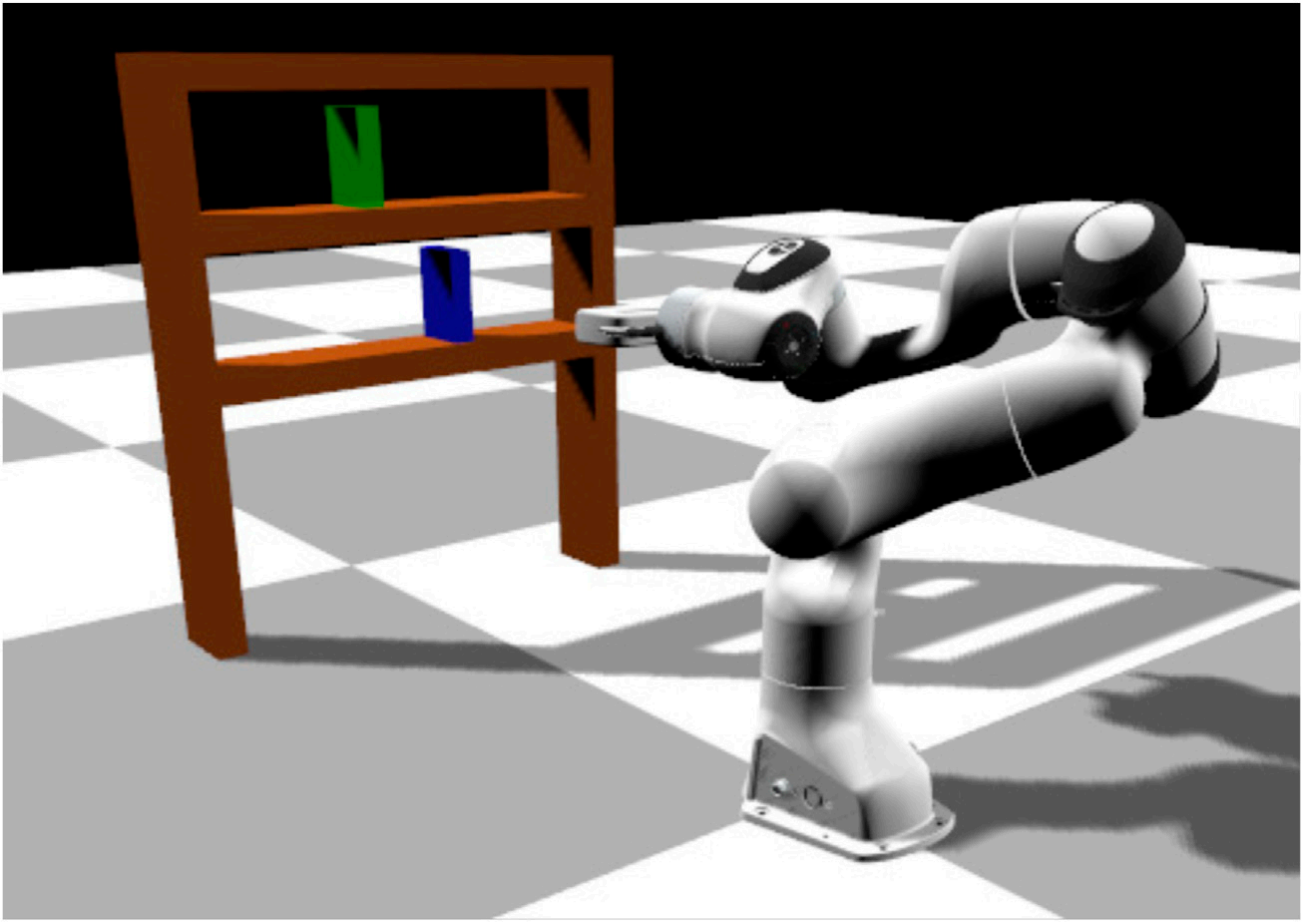
Bookcase task: the robot needs to grasp a book (in blue) and place it in a goal position (in green).

In this scenario the environment consists of the same manipulator, a bookcase with two shelves, a book (blue color in Figure 4) and a goal position (green color in Figure 4). The book is initially placed in the bottom shelf and the manipulator has to move it to the goal in the upper shelf. The initial position of the book vary but the goal position is kept fixed. In this task, the manipulator has to perform a longer and more precise sequence of sub-tasks: 1) go to the book, 2) grasp the book, 3) lift it slightly up, 4) pull it out of the bookcase, 5) go to the upper shelf and 6) place the book in the goal position. We provide limited results on this task, and we do not vary the goal location.

### 3.2 Imitation learning evaluation

**FIGURE 5:**
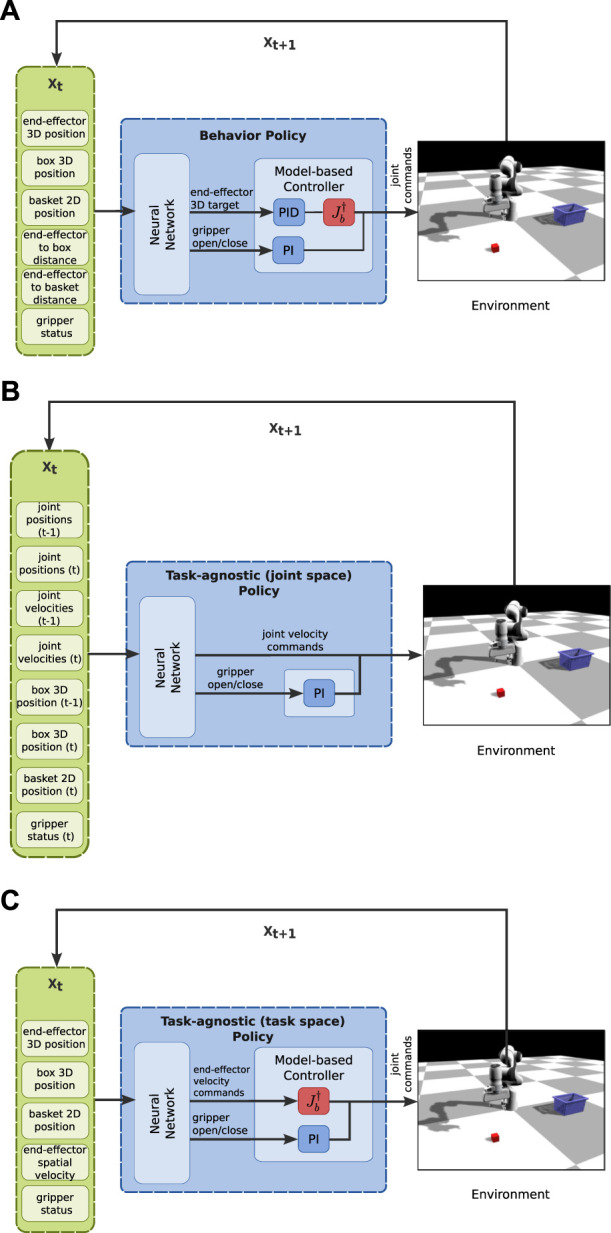
Structure of each policy for the pick and place task. **(A)** The structure of the proposed BPL for the pick and place task. The task-specific policy has also the same structure, but the neural network outputs the next 3D target for the end-effector to complete the current stage of the task as provided by the FSM. **(B)** The structure of the task-agnostic (joint space) policy. **(C)** The structure of task-agnostic (task space) policy.

**FIGURE 6:**
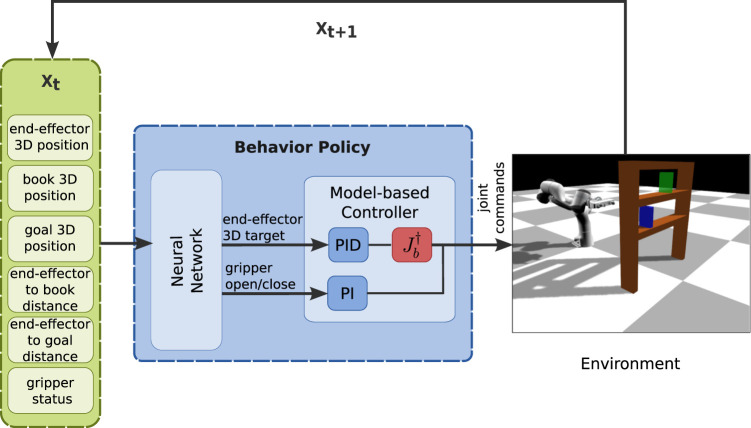
The structure of the proposed BPL for the bookcase task.

In this section, we aim at answering the first three questions of Section 3. Thus, we utilize the two tasks and use only the learning from solution sketches part of our method. We collect one dataset and perform *10 independent supervised learning trainings with different seeds*. The neural network part of the BPL accepts as input the state of the robot (end-effector position and gripper status), the state of the environment (position of objects of interest), and generic environment-specific information (distance of end-effector to key objects/states). We compare our BPL (Figures 5A, 6) to the following policy structures:

• A policy structure that has the same inputs/outputs with the BPL but is trained to predict the next target provided from the hard-coded FSM (an expert policy): this policy serves as a baseline that uses extensive task knowledge (we refer to it as *task-specific*, Figure 5A);
• A policy structure with inputs the state of the robot and the environment (no distances to objects), but that has access to the control commands in joint space (i.e., the neural network directly predicts the next velocity commands for the joints): this policy is the traditional “Behavior Cloning” policy in the literature (we refer to it as *task-agnostic (joint space)*, Figure 5B));
• A policy structure with inputs the state of the robot and the environment, but that has access to the control commands in end-effector space (i.e., the neural network directly predicts the next velocity commands for the end-effector): this policy is similar to the previous one, but uses the pseudo-inverse of the jacobian of the end-effector to transform the commands from end-effector space to joint space (we refer to it as *task-agnostic (task space)*, Figure 5C)).

#### 3.2.1 Data collection and preprocessing

We collect the solution sketches in a dynamical simulator by recording only the state variables while completing the task using a hard-coded Finite State Machine (FSM) and a model-based controller in end-effector space that operate at 40 Hz. During the demonstrations collection, we vary the positions of the task-related objects in the environment, but we keep the orientations fixed.

For the pick and place task, the 3D positions of the box and the basket are sampled uniformly and their orientation is kept constant. To generate episodes with different combinations of the 3D positions of the environment objects, we perform a Centroidal Voronoi Tesselation (CVT) on a large number of points, i.e. 100 k, that have been sampled uniformly on the 3D space of the polar coordinates of the box and the basket (*r*_*box*_, *θ*_*box*_ and *r*_*basket*_). In addition, for each box position we generate the episode twice with the basket placed on each side of the manipulator. For the bookcase task, we vary only the position of the book in the bottom shelf. The goal position is in the upper shelf and remains the same in each episode. We provide preliminary results for the bookcase task, and more advanced versions of this task are to be examined in future work. The initial configuration of the manipulator and the positions of the environment objects are specified in Table 1 for both tasks. We generate more episodes, i.e. 50, to collect solution sketches for the pick and place task as we vary more parameters of the environment and only 10 for the bookcase task.

**TABLE 1:** Environmental setup used during data collection.


Pick and place task	Robotic manipulator initial configuration (joint positions for each DoF in radians)	A1: 0, A2: 0, A3: 0, A4: − *π*/2, A5: 0, A6: *π*/2, A7: *π*/4
	gripper initial configuration (m)	0.04
	robotic manipulator position (m)	x: 0.0, y: 0.0, z: 0.0
	box position in polar coordinates *r* (m), *θ* (rad)	r:U(0.4,0.65)
		θ:U(−π/4,π/4)
	basket position in polar coordinates *r* (m), *θ* (rad) (we use *θ*_1_ in the first half of the episodes and *θ*_2_ in the second one)	r:U(0.45,0.65)
		$\theta_{1}:U\left(-\pi /2,-\pi /3\right)$
		$\theta_{2}:U\left(\pi /3,\pi /2\right)$
Bookcase task	robotic manipulator initial configuration (joint positions for each DoF in radians)	A1: 0.58747083, A2: − 1.19088859, A3: − 0.95177053, A4: − 2.81123903, A5: − 2.89731855, A6: 2.78075701, A7: 2.8726956
	gripper initial configuration (m)	0.04
	robotic manipulator position (m)	x: 0.0, y: 0.0, z: 0.0
	bookcase position (m)	x: 0.8, y: 0.0, z: 0.0
		bottom shelf (z): 0.34
		upper shelf (z): 0.52
	book position (m)	y: 10 evenly spaced numbers in the interval [ − 0.18, 0.18] on the bottom shelf
	goal position (m)	y: 0.05 on the upper shelf

In order to facilitate training and learn optimal policies for each task, we take advantage of the full ground truth information of the simulated environment. Thus, at each time step of an episode we record proprioceptive data about the configuration of the robotic manipulator, i.e. the joint positions and velocities, the end-effector position and velocity, and information about the other objects of the environment, i.e. position of the box and the basket without using a perception module (we use a perception module in evaluation phase). For the task-agnostic policies, we collect demonstrations that also include the control commands. As a preprocessing step, we standardize each column of the input vectors.

#### 3.2.2 Neural network architecture

The neural network architecture of the proposed BPL for the pick and place and bookcase task is specified in Tables 2, 3 respectively. In addition, for the first task we specify the network architectures of the task-specific and task-agnostic policies to which our proposed policy is compared.

**TABLE 2:** Policy network architecture for the pick and place task.

Behavior	Task-specific	Task-agnostic (task space)	Task-agnostic (joint space)
Input (11)	Input (11)	Input (15)	Input (40)
Dense (16)	Dense (32)	Dense (128)	Dense (128)
Tanh()	Tanh()	Tanh()	Tanh()
Dense (16)	Dense (32)	Dense (128)	Dense (128)
Tanh()	Tanh()	Tanh()	Tanh()
Dense (4)	Dense (4)	Dense (64)	Dense (128)
[Linear(3),Sigmoid(1)]	[Linear(3),Sigmoid(1)]	Tanh()	Tanh()
		Dense (7)	Dense (8)
		[Linear(6),Sigmoid(1)]	[Linear(7),Sigmoid(1)]

**TABLE 3:** Policy network architecture for the bookcase task.

Behavior
Input (12)
Dense (32)
Tanh()
Dense (32)
Tanh()
Dense (4)
[Linear(3),Sigmoid(1)]

##### 3.2.2.1 Pick and place task

The input vector to the behavior and task-specific policies consists of: 3D position of the end-effector, 3D position of the box, 2D position of the basket (we assume that the z-axis coordinate is not important for training the network), end-effector to box distance, end-effector to basket distance and joint position of the gripper. These two policies output the next 3D position of the end-effector and a probability of the next state of the gripper (open or close). The difference is that the proposed BPL is trained to output the 3D target of the end-effector *k* time-steps in the future, whereas the task-specific is trained to output the 3D target of the end-effector for each stage of the task as provided by an FSM. The task-agnostic (task space) policy network takes as input the same 3D positions of the end-effector, box and basket and the joint position of the gripper as in the previous policies and the end-effector spatial velocity. Instead of the 3D target position of the end-effector, this network is trained to output the end-effector velocity commands that move the end-effector to the target position. Finally, the task-agnostic (joint space) policy takes as input the robot joint positions and velocities of the previous and current time-step, the 3D position of the box at the previous and current time-step and the 2D position of the basket. The output vector consists of velocity commands for each controllable DoF and the gripper command. We use a larger network to represent the task-agnostic policies due to the complexity of the function they have to approximate. For the task-agnostic policy in joint space, we used the state of the environment of the previous timestep as well since in preliminary experiments, this was working better.

##### 3.2.2.2 Bookcase task

The BPL network takes as input the 3D positions of the end-effector, book and goal and the gripper status and outputs the 3D target position of the end-effector and the gripper command as in the first task.

##### 3.2.2.3 Neural network hyperparameters

The values of the hyperparameters we use for training the neural networks are specified in Table 4. For the networks of the task-agnostic policies, specifically, we use a scheduler to adjust the learning rate during training: starting from the value of 3e − 4 the learning rate decays if the loss in the validation dataset is not improved for 10 epochs.

**TABLE 4:** Training hyperparameters.

Task	Policy	Batch size	Optimizer	Epochs
Pick and place	Behavior	256	Adam(3e-4)	600
	Task-specific	256	Adam(3e-4)	600
	Task-agnostic (task space)	256	Adam(3e-4)	1800
	Task-agnostic (joint space)	256	Adam(3e-4)	1800
Bookcase	Behavior	128	Adam(3e-4)	1,500

The loss function $L_{\mathrm{total}}$ (Eq. 3) that is used for training the network of the proposed BPL is a combination of the mean squared error of the 3D target position of the end-effector (*MSE*_*T*_), the binary cross entropy loss of the gripper command (*BCE*_*G*_) and a *L*_1_ penalty term of the weights of the network *w*.

$$L_{\mathrm{total}}=\alpha MSE_{T}+BCE_{G}+\lambda \overset{m}{\underset{i=1}{\sum }}|w_{i}|$$ (3)

We observed that the binary cross entropy loss of the gripper command is much greater than the mean squared error of the 3D target. Thus, we put a big value to the weight *α* to impose a larger penalty to the target loss. In addition, the regularization weight *λ* is set to 1e − 3. In the task-agnostic policies, specifically, we noticed that the network could not learn (loss was high and not decreasing) with this value of the regularization weight *λ*, but only with a very small value, i.e. 1e − 6. Thus, we decided not to use a *L*_1_ penalty term at all during the training of the task-agnostic policies.

#### 3.2.3 Imitation learning results

##### 3.2.3.1 Pick and Place task

**FIGURE 7:**
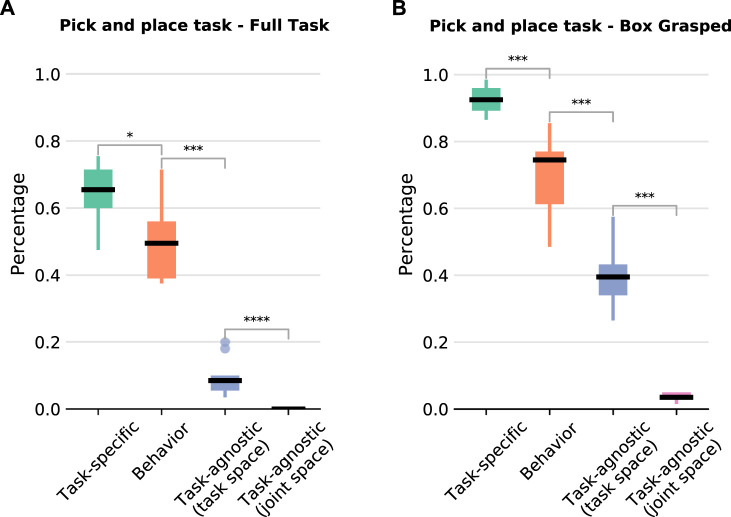
Pick and place task results over 10 independent trainings. The number of stars indicates that the *p*-value of the Mann-Whitney U test is less than 0.05, 0.01, 0.001, and 0.0001 respectively. **(A)** Percentage of episodes completing the full task (grasp the box and place it inside the basket) for each policy structure. **(B)** Percentage of episodes in which the box is successfully grasped but it was not placed inside the basket.

For this task, as there are many variations in the initial poses of the box and the basket, we collect *50 solution sketches*. We evaluate the learned policies with 112 different initial configurations (see Figure 3). The results showcase that our BPL is comparable to the policy that uses extensive task knowledge and much better than the other baselines (Figure 7A). Overall, our BPL achieves a median success rate of 50% over 10 independent trainings, while the task-agnostic policies almost completely fail to achieve the task (medians of 9% and 0% for end-effector and joint space respectively). The task specific policy has the best performance and achieves a median success rate of 66%. Moreover, our policy grasps box 75% of the time even if it fails to complete the task, whereas the agnostic policies grasp it less than 40% of the time; the task-specific policy grasps it 93% of the time (Figure 7B).

##### 3.2.3.2 Bookcase task

**FIGURE 8:**
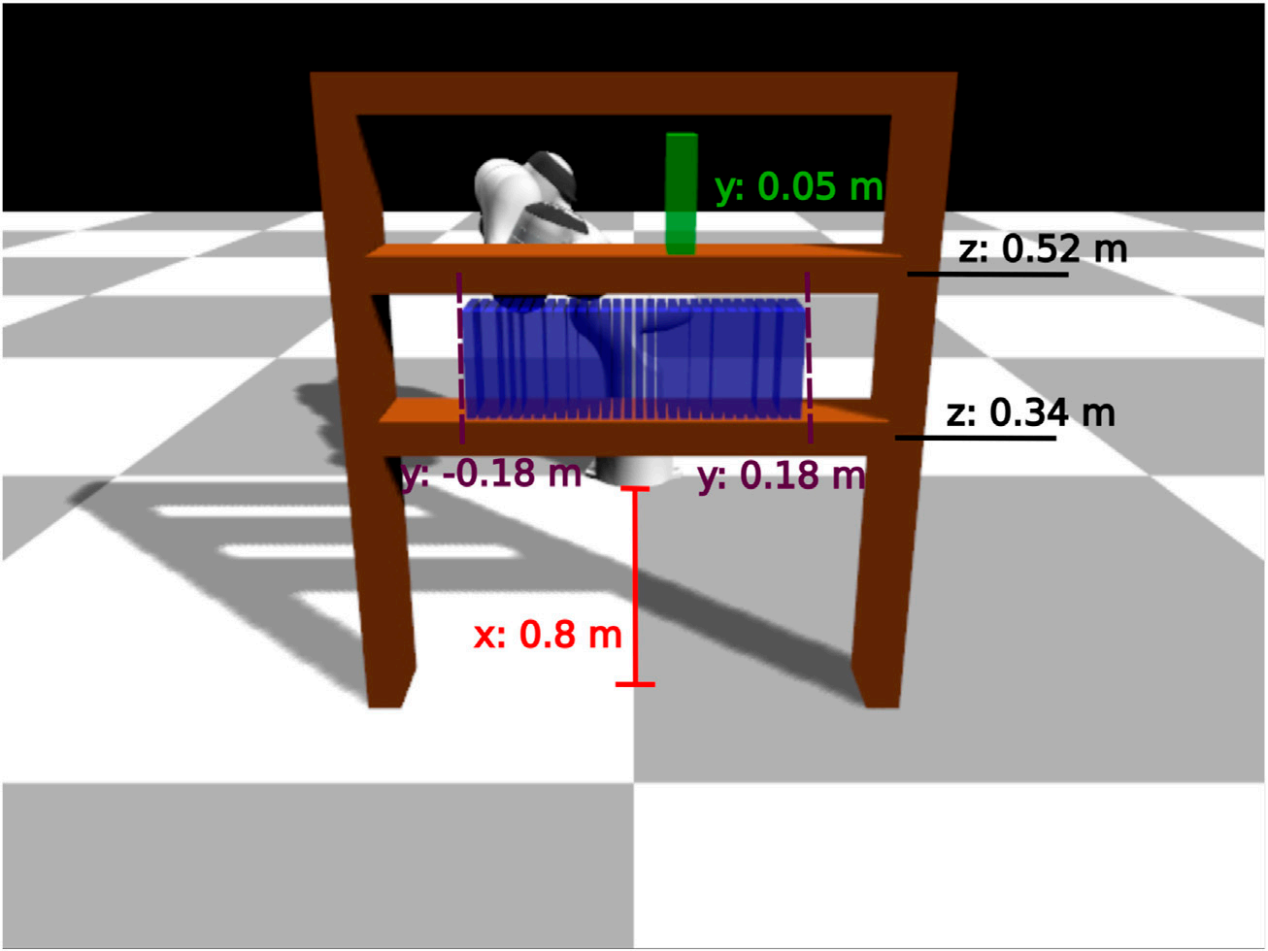
Bookcase task: evaluation configurations. We use 30 different initial book positions (denoted with blue color).

Since we only alter the initial position of the book on the shelf, we managed to produce very good results with as few as *10 solution sketches*. We evaluate the learned policies using 30 different initial book positions, evenly spaced at the bottom shelf (Figure 8). In this task, an episode is considered successful if a book is placed at a maximum distance of 5 *cm* from the goal position and the end-effector’s distance from the final position of the book is at least 0.3 m for 10 consecutive time-steps. Our BPL managed to achieve a median of 85% success rate over 10 independent trainings, while the task-agnostic policies did not manage to solve the task (less than 5% success rate). We also struggled to make the task specific policy to work reliably: possibly because the FSM for this task was more complex.

### 3.3 Behavior policy learning evaluation

In order to answer to the last question of Section 3 and evaluate the full BPL pipeline in simulation, we devise a realistic version of the pick and place task, where the objects are tracked through an RGB-D camera and no ground truth information is given to the robot. The visual sensor operates at a rate of 20 Hz to emulate the typical frequency of real camera sensors and mismatch of control and sensor frequencies. We have implemented a perception module where: 1) a point cloud is extracted from the depth map generated by the calibrated RGB-D sensor, 2) the points which lie on the floor and the body of the robot are filtered out and 3) the remaining ones are clustered and the object positions are determined from the medians of the two largest clusters. We provide preliminary results, and take the best three policies from the imitation learning step and further fine-tune them as described in Section 2.4. The results show that we achieve an *average improvement of 23%* and the optimized policies have a *median success rate of 76%* (see Table 5).

**TABLE 5:** Policy Fine-Tuning Results (the best three policies from the imitation learning step).

	Success rate (with perception module)	
	Before fine-tuning (%)	After fine-tuning (%)
Policy #1	66	75
Policy #2	64	82
Policy #3	60	76

### 3.4 Physical robot results

**FIGURE 9:**
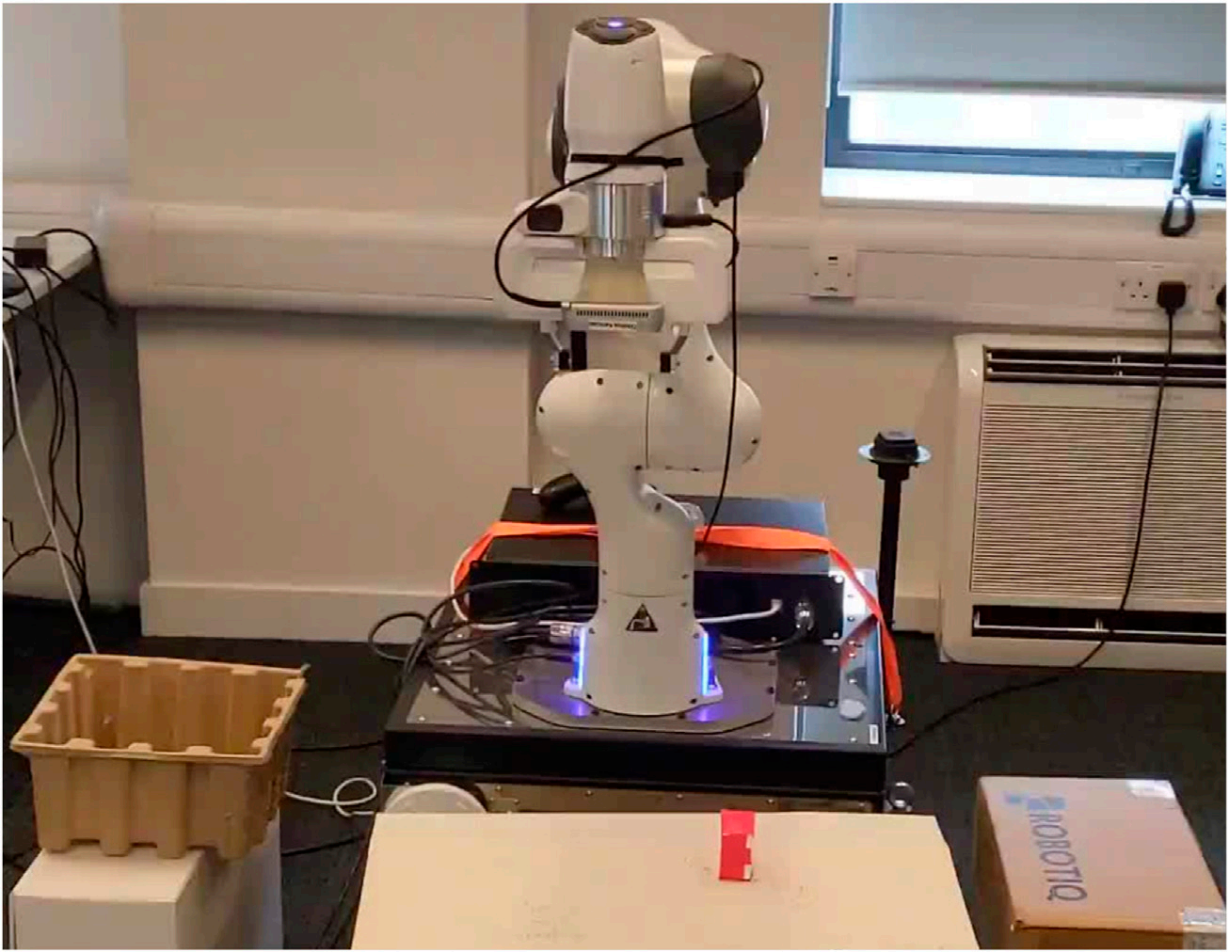
Physical robot setup: goal object to pick is in red, while the goal place is the box on the left.

**FIGURE 10:**
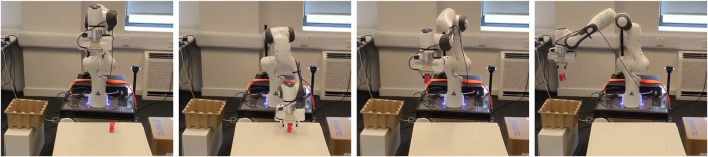
BPL successful implementation on the real setup (times advances from left to right). The policy learned in simulation is able to achieve 67% success rate on the physical setup despite the reality gap and mismatches on the low-level controller.

In order to answer to the last question of Section 3, we also provide preliminary results on a physical setting of the pick and place task (Figure 9). We take one of the optimized policies of the previous step (the complete BPL pipeline) and apply it on the real robot. We evaluated the policy on 12 different initial configurations (6 positions of the box for 2 different positions of the basket). Our method achieves 67% success rate (8 out of 12) despite the fact that we were using a different low-level controller, i.e. impedance Cartesian controller, and the reality gap. Figure 10 shows a policy execution on the physical setup.

## 4 Discussion and conclusion

Our BPL pipeline makes it possible to learn effective policies for multi-stage tasks by utilizing few demonstrations and fine-tuning in simulation. BPL also lifts the need of having demonstrations with optimal actions, and only requires the state variables. We demonstrated the effectiveness of our proposed policy structure and pipeline in two different tasks in realistic simulations. We also provided preliminary results on a physical setting.

Our policy is inspired from the LfD literature, and we attempted to make the underlying policy structure more flexible and easier to fine-tune with RL. Structured policies have been discussed and analyzed in depth in the literature (Stulp and Sigaud, 2013; Martín-Martín et al., 2019; Varin et al., 2019), and we see our work as further validation that the type of structure of a policy is crucial, and as an analysis of the key parts of a policy for effective LfD. In essence, our work provides a practical approach for learning only from a few solution sketches, while utilizing useful and generic task-agnostic information from the task (e.g., model of the robot, distances to objects, etc.).

Moreover, many approaches have been proposed for combining demonstrations in a policy search RL setting (e.g. (Rajeswaran et al., 2018; Zhu et al., 2018; Thananjeyan et al., 2020)) with pipelines similar to our method. There are three key differences compared to our work: 1) we do not propose a novel learning method for any policy structure, but rather a practical pipeline and specific structured policy for effectively learning from very few demonstrations, 2) we require very few solution sketches, whereas the proposed methods in the literature usually need at least 100 full demonstrations (with action commands), and 3) the policies learned from the imitation (or behavior cloning) part of our method are already quite effective and thus require only small fine-tuning with RL afterwards.

Here it is important to note that we consider the first part of our approach being a pure imitation learning method that suffers from all the well-known issues of BC. The most important limitation is the well-known distribution shift (or covariate shift) Osa et al. (2018); Bagnell (2015); Ravichandar et al. (2020); in short, as the set of demonstrations is small, the test distribution (i.e., the actual running of the learned policy) can be–and usually is–different from the demonstrated conditions. This leads to great deterioration of performance since the small errors per step are compounding errors that eventually lead the behavior in a space completely outside of the train distribution. This is usually tackled with interaction with the system (applying also algorithms for effective online demonstrations Bagnell (2015)), stricter assumptions about the system (i.e., we know more about the system) Billard et al. (2008), and/or extensive domain randomization Rudin et al. (2022); Lee et al. (2020).

Another important thing to note is the fact that we view our proposed policy structure as an effort to bridge the more traditional LfD literature (Billard et al., 2008) with more recent methods from the RL literature (Rajeswaran et al., 2018; Gupta et al., 2019). In this view, we began from the LfD literature (by creating a policy structure that encodes a trajectory) and attempted to provide more flexibility so that we can take advantage (in future work) of effective RL toolkit.

Even though using our BPL pipeline the learned policies were effective, we provide no theoretical guarantees for stability, which is important for robotic applications. In future work, we will attempt to merge neural networks with dynamical systems in order to get the best of both worlds. In (Bahl et al., 2020), a method to combine neural networks with dynamical systems has been recently proposed, but since their policy changes the dynamical system every *N* steps there are still no theoretical guarantees for stability. We aim at filling this gap.

Our approach relies on model-based object tracking (*via* an RGB-D camera), and this can be difficult to have for any object. In future work, we aim at defining object-agnostic structures (e.g., 3D voxels) to be used as inputs to our final policy, and trained in simulation *via* privileged learning (see for example (Hwangbo et al., 2019)). Finally, in future work we will use state-of-the-art RL methods for the policy fine-tuning part and perform more extensive evaluations both in simulation and the physical world.

## Data Availability

The raw data supporting the conclusion of this article will be made available by the authors, without undue reservation.
